# Potential Applications of *Lilium* Plants in Cosmetics: A Comprehensive Review Based on Research Papers and Patents

**DOI:** 10.3390/antiox11081458

**Published:** 2022-07-27

**Authors:** Yuchao Tang, Yijie Liu, Kang Luo, Leifeng Xu, Panpan Yang, Jun Ming

**Affiliations:** 1 Institute of Vegetables and Flowers, Chinese Academy of Agricultural Sciences, Beijing 100081, China; tangyuchao100@126.com (Y.T.); liuyijie1209@163.com (Y.L.); lk1689252548@163.com (K.L.); xuleifeng@caas.cn (L.X.); yangpanpan@caas.cn (P.Y.); 2 College of Landscape Architecture and Forestry, Qingdao Agricultural University, Qingdao 266109, China

**Keywords:** *Lilium* plant, polyphenols, polysaccharides, cosmetic application, antiaging, ultraviolet blocker, skin whitening, moisturizing, freckle removal, acne treatment

## Abstract

The application of cosmetics is indispensable in our current society. In recent years, with an increasing awareness of the long-term health benefits of naturally sourced ingredients, plant-based cosmetic products have gained increasing attention. *Lilium* belongs to the Liliaceae family, which is one of the main plant families used in cosmetics for skin care treatment. A large number of studies have shown that *Lilium* plants are rich in components such as phenolic acids, flavonoids, and polysaccharides, with high potential for cosmetic applications. However, the application of lilies in cosmetics has not been systematically reported. This knowledge gap can easily lead to the neglect of its application in cosmetics because lilies are most familiar as ornamental plants. Integrating academic papers and patent publications, we analyzed the potential cosmetic application ingredients in lily, as well as their applications in cosmetics and related efficacy. Patent analysis showed that applications for lily-related cosmetic patents are mainly concentrated in East Asia, including China, Korea, and Japan. The application of lilies involves all aspects of cosmetics, such as sunscreens, facial cleansers, facial masks, conditioners, and so on. Its functions are also rich and diverse, including antiaging, radiation protective, whitening, moisturizing, freckle removal, acne treatment, and hair regeneration promotion. In addition, lilies are compatible with the application of other herbs. Moreover, with a change in people’s consumption concepts and the consideration of long-term health benefits, lily-based food and medicine innovation with health care and beautification effects may be a promising direction.

## 1. Introduction

The application of cosmetics has been an essential part of our lives since ancient times. Archaeological study has shown that cosmetics were used in ancient Egypt from approximately 3000 B.C. [1], and their uses in China can also date back to approximately 2700 years ago [2]. Nowadays, the cosmetic industry is thriving. The global cosmetics industry was valued at more than $500 billion in 2017 and is growing rapidly every year [2]. The consumption concept of “green beauty” also promotes a tendency to purchase cosmetic products of natural origin [3]. Moreover, plant-derived cosmeceutical formulations are also gaining popularity because of their long-term health and cosmetic benefits [4]. Plant-based natural products are wildly used in cosmetic compositions for skin care, hair care, and toiletry preparations because they are generally considered safe and do not predispose consumers to allergic reactions [1,5,6,7]. Beauty-related cosmetics of plant extracts are due to their antioxidant, antibacterial, wound-healing, antiaging, sun protection, cytoprotective, skin whitening, and anti-inflammatory activities [4]. Therefore, it is of great scientific significance and production value to fully evaluate the use of plants, especially medicinal and edible plants rich in active ingredients, in cosmetic products.

*Lilium* plants are widely cultivated worldwide due to their outstanding ornamental, edible, and medicinal value. The genus *Lilium* belongs to the family Liliaceae, which is one of the major plant families used in cosmetic formulations for skin care [8]. Many studies have shown that *Lilium* is rich in amino acids; polysaccharides; and bioactive components such as phenolics, flavonoids, and saponins, which are important sources of natural products [9,10,11,12,13]. These compounds have proven to exert numerous health benefits, such as anti-inflammatory [14], antitumor [15], hypoglycemic [16,17,18], and antidepressant [19] effects, as well as ultraviolet (UV) absorption, free radical scavenging, carcinogenesis inhibition, wrinkling, and other skin care effects [4,5,20]. Antioxidant and ultraviolet absorption capacities are important evaluation indexes for the superiority and inferiority of cosmetic products [21]. Polyphenols (including phenolic acids, flavonoids, lignin, etc.) have good UV absorption abilities because they contain aromatic rings [22]. Our previous studies showed that both phenolic acids and flavonoids are abundant in the lily bulbs of different genetic backgrounds [12]. Moreover, phenolic hydroxyl groups have a strong antioxidant capacity [23]. These results illustrate that lilies have great potential for cosmetic applications.

In fact, although rare, some investigators have reported the application of lilies in cosmetics. *Lilium candidium* is one of the most commonly used herbs for the treatment of freckles [20]. The associated compound contained in *L. candidium* can relieve periorbial hyperchromia (dark circle) symptoms [24]. The emulsion containing the extract of lily flowers was shown to be effective in promoting skin regeneration, leading to cosmetic results [25]. According to the catalog of applied cosmetics raw materials (2021) compiled by China Food and Drug Administration, the cosmetic products using the extracts of *L. candidum*, *Lilium lancifolium*, and *Lilium japonicum* and the flower oil of *Lilium brownii* have obtained the approval of imported/domestic special/non special purpose items [26].

However, although there are a large number of research papers on the chemical composition of *Lilium* spp., only a few academic papers have mentioned their usages in cosmetics. In contrast, there are thousands of cosmetic products claimed to have taken lily extracts as an added ingredient (https://www.bevol.cn/index.html) (accessed on 20 January 2022). Patent publications, extensively used in exploring technological innovation and evolution, are important scientific references [27]. These patents contain rich information and new results and are usually considered the appropriate data for analyzing scientific outcomes [28,29]. Related data showed that 56.6–94% of the contents reported in patents in different fields worldwide have never been published in any other way, including in scientific research papers. Even if some patents are published as papers, the time of their publication will lag by 1.7–3.7 years [30]. Therefore, in this review, (1) the chemical constituents of *Lilium* with potential cosmetic applications were summarized; (2) based on the patent, the application field, main efficacy, and compatible ingredients of lilies in cosmetics were analyzed; and (3) the opportunities, challenges, and development potential of *Lilium* in cosmetics applications were discussed.

## 2. Botany of *Lilium*

The genus *Lilium* is a perennial herbaceous bulbous plant that belongs to the family Liliaceae, containing about 115 wild species and thousands of hybrids [31]. Because of their prominent ornamental, edible, and medicinal values, lilies are widely cultivated worldwide. According to their morphological characteristics and origins, lilies are classified into 7 sections, i.e., section *Pseudolirium*, section *Liriotypus*, section *Archelirion*, section *Sinomartagon*, section *Leucolirion*, and section *Daurolirion* [32]. After decades of hybrid breeding by breeders, 7 main groups of cultivars have been bred: O (Oriental hybrids), A (Asiatic hybrids), L (*L. longiflorum* hybrids), LO (*L. longiflorum* × Oriental hybrids), LA (*L. longiflorum* × Asiatic hybrids), T (Trumpet hybrids), and OT (Oriental × Trumpet hybrids) [33].

**Figure 1:**
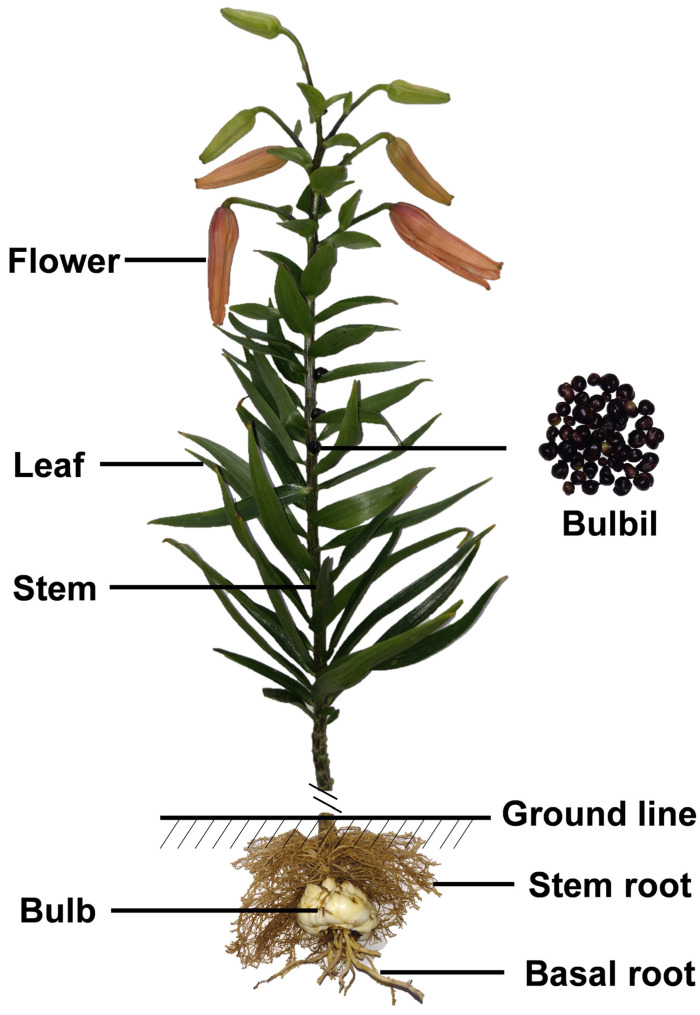
Whole plant of Lilium lancifolium. Note: the bulbil, as a special axillary vege tative reproductive organ, is only naturally produced in four wild lilies (*L. lancifolium*, *L. sulphureum*, *L. sargentiae*, and *L. bulbiferum*) and some A and LA hybrids.

Taking *L. lancifolium* as an example, the complete plant architecture of a lily is shown in Figure 1. A bulbil, as a special axillary vegetative reproductive organ, is produced only in four wild lilies (*L. lancifolium*, *L. sulphureum*, *L. sargentiae*, and *L. bulbiferum*) and some A and LA hybrids [33,34]. In most cases, lilies are well known as ornamental plants, for cutting flowers, potting, flower landscapes, or home gardening. In fact, many lilies are used as food and in medicine, worldwide. It has been reported that more than 30 *Lilium* species have long been consumed as food or used for disease treatment in different countries because of their strong antioxidant and UV-blocking ability [12,25,35]. According to traditional Chinese medicine (TCM), *L. lancifolium* Thunb., *Lilium brownii* F. E. Brown var. *viridulum* Baker, and *Lilium pumilum* DC. are used medicinally for disease treatment [36]. Moreover, lily extracts are also used as beautifying substances in a variety of cosmetics [20,24,25].

## 3. Ingredients with Potential Application in Cosmetics in *Lilium*

The skin is the largest organ of the human body and is often directly exposed to environmental conditions and highly susceptible to damage by hazardous substances and sunlight [37]. Excessive solar exposure, especially strong ultraviolet (UV) radiation, can easily lead to skin aging and lesions [38,39,40]. Therefore, compounds with photoprotective activity are extremely useful to reduce the damage to the skin by UV radiation [21]. However, many sun blockers, especially organic sunscreens, often cause allergies [41,42]. Plant products have long been important sources of food and medicine. The function of a plant is mainly due to its chemical composition [43]. Among these compounds, phenolics have attracted much attention in cosmetics because of their outstanding antioxidant, bacteriostatic, antiaging, and skin-repair functions [44]. Because of the aromatic ring, phenols have a strong absorption capacity of 200–400 nm light waves, so they are popular natural UV blockers [21]. In plants, phenolic acids are mainly produced by the shikimate pathway [45]. The two principal skeletons of phenolic acids in plants are C6–C1 and C6–C3. The former has a hydroxybenzoic acid skeleton and includes protocatechuic, gentisic, gallic, vanillic, and syringic acids. Additionally, C6–C3 phenolic acids have a hydroxycinnamic acid skeleton and include caffeic, sinapic, p-coumaric, and ferulic acid. [46]. Under the catalysis of a series of enzymes, phenolic acids, flavonoids, tannins, and anthocyanins are formed [47].

Studies have suggested that phenols are one of the most abundant bioactive components in lilies [9,11,12,13]. Phenolic acids and flavonoids are the most abundant polyphenols in lilies. In our previous study, 153 phenolic acids and 201 flavonoids were detected from different lilies, accounting for more than half of the total number of detected secondary metabolites [12]. Table 1 presents the total phenolic acid content (TPC) and total flavonoid content (TFC) in different lily materials, from which we can see clearly that TPC and TFC vary greatly among different *Lilium* bulbs, even more than 10 times (Table 1). In some lily species that are not developed for food, drug, or cosmetic applications (e.g., *L. henryi*, *L. leucanthum*, *L. pumilum*, *L. regale*, *L. rosthornii*, *L. sargentiae*, *L. sulphureum*, and *L. taliense*), the TPC and TFC are significantly higher than in species that have been widely used (e.g., *L. brownii*, *L. brownii* var. *viridulum*, *L. davidii* var. *unicolor*, *L. davidii* var. *willmottiae*, *L. lancifolium*, and *L. pumilum*) [9]. Moreover, the TPC and TFC are also much higher in many cultivars than in the widely used lily species [12]. The TPC in lily bulbs is much higher than that in some leafy vegetables such as *Brassica rapa* L. ssp and *Amaranthus*, and some wild fruits such as *Celtis australis*, *Ficus palmata*, *Morus alba*, and *Prunus armeniaca* [9]. Additionally, the TFC in some lilies was higher than that in *B. rapa* and *Vitis davidii* [9]. In addition, our latest study has also shown that the total phenolic acids and total flavonoids in the aerial parts of lily plants are significantly higher than those in the underground parts of scales, implying that the stems, leaves, and flowers of lilies also have potential cosmetic uses (unpublished).

Antioxidation is also an important embodiment of the skin care function of polyphenolic ingredients. Strong UV radiation excites the production of hydroxyl radicals, singlet oxygen, hydrogen peroxide, and superoxide anions to cause myosomal oxidative stress disorders, which induce skin diseases [49,50]. Because phenolics usually have a large conjugated structure, which makes the phenoxyl radicals formed after producing a hydrogen atom highly stable and hardly reactive, they are excellent natural antioxidants [23]. In vitro antioxidant assays showed that different lily extracts have strong antioxidant capacity, including DPPH radical scavenging ability, ABTS radical scavenging ability, copper-ion-reducing ability, and ferric ion reducing ability (Table 1) [9,12,13]. Antioxidant capacity has significant positive correlations (r > 0.98, *p* < 0.01) with phenolic acid and flavonoid contents [12]. In vivo, phenolic acid components such as cinnamic acid can be directly absorbed by the human body, while flavonoids are broken down into simple phenolic acids in the digestive system and then enter the blood circulatory system for transport to various parts of the body [51]. It has been shown that the polyphenol-rich lily extract had good efficacy for skin care, whether it was blocking UV radiation from the exterior or maintaining oxidative stress balance from the interior.

Polysaccharides are among the most abundant carbohydrates in plants [37]. Studies have shown that plant-derived polysaccharides have many potential biological activities, such as bacteriostasis; the promotion of wound healing; and antioxidation, antitumor, anti-inflammatory, and antiaging activities [37]. Polysaccharides contain many hydroxyls and polar groups, which can form hydrogen bonds with water molecules, leading to strong water retention characteristics [52,53,54]. Polysaccharides are one of the richest carbohydrate components in a lily, accounting for 10–36% of the bulb dry weight [55,56,57]. Thus, lily polysaccharides are a good natural ingredient for skin moisturizing products. Moreover, lily polysaccharides also exhibited antioxidant, bacteriostatic, anti-inflammatory, wound-repair, and cancer-cell inhibition efficacy, indicating their potential application in skin repair and the inhibition of skin carcinogenesis [16,56,57,58,59,60].

Moreover, carotenoids and anthocyanins are also great antioxidants and ultraviolet absorbers [21]. Different lily bulbs contain 0.13~6.63 (mg/100 g DW) carotenoids and 0.64~9.63 (Cyanidin-3-O-glucoside equivalent mg/100 g DW) anthocyanins [9]. Furthermore, there are abundant components such as amino acids, minerals, saponins, and alkaloids that are beneficial to the skin [9,35]. The available studies have shown the great potential of lilies in cosmetic applications. However, the research on their specific application in cosmetics and their mechanism of action, such as that in skin care, still needs to be strengthened.

## 4. Patents of Lily-Based Cosmetics (2000–2021)

### 4.1. Acquisition of Patent Data

As a reliable source of patent data, PatSnap (https://www.patsnap.com/) (accessed on 28 January 2022) provides more than 170 million patent documents covering 158 countries, including World Intellectual Property (WIPO) and the European Patent Office (EPO) [61]. To gain further insight into the use of the *Lilium* plant in cosmetics, we retrieved all *Lilium*-related cosmetic patents utilizing PatSnap (accessed on 28 January 2022). We use the process of preliminary search → determination of search formula → accurate search → de-duplication by family → manual screening to search for lily-related cosmetics patents. After several attempts, we retrieved “((Lilium OR lily) NOT (Day Lily OR Day-lily OR Fan Lily OR Fan-lily OR Water Lily OR Water-Lily OR lily of the valley)) AND ((Cosmetic) OR (Cosmetics) OR (soap) OR (toothpaste) OR (Facial mask) OR (Toner) OR (sunscreen) OR (whiten) OR (whitening) OR (spot-reducing) OR (spot reducing) OR (lighting) OR (light) OR (shampoo) OR (softener) OR (detergent) OR (pre-shave) OR (nourishing) OR (freckle) OR (moisturizing) OR (rejuvenating))” as the search formula. The time range was set from 2000 to 2021. Entries with legal status withdrawn, ceased, expired, abandoned, revoked, rejection, and discontinuation were excluded. After the manual removal of unrelated patents, we obtained patents published in English or accurately translated into English for further analysis.

### 4.2. Overview of Lily-Based Cosmetics Patents

**Figure 2:**
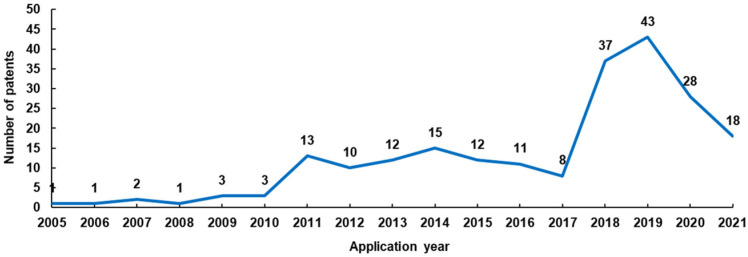
Number of lily-related cosmetics patents applied for in different years.

After screening, we obtained 218 patents for follow-up analysis. The screened patent data, including the publication number, application date, publication date, legal status and events, international patent classification (IPC), first inventor, original assignee (applicant), original assignee address, and simple family, were imported into Excel for analysis (Supplementary Table S1). In terms of legal status, 84 patents are under examination, 102 patents were granted, 36 patents have been transferred (one of which is under examination), 31 patents are only in publication status, and the legal status of one patent is unknown (Supplementary Table S1). From the perspective of application year, the number of lily-related patent applications has experienced two stages of rapid growth. Before 2010, there were few patent applications for lily-related cosmetics, all of which were less than five. This number increased rapidly in 2011, with more than 10 applications per year until 2016. In 2018, the number of patent applications soared again, reaching 37 in 2018 and 43 in 2019. Up to 28 January 2022, we retrieved 28 and 18 lily-related cosmetics patent applications in 2020 and 2021, respectively (Figure 2). The decline at this stage may be due to the lag in patent publication time (Supplementary Table S1). These data indicate that patent applications for lily-related cosmetics are in a prosperous period, implying that the application of lilies in cosmetics is gaining increasing attention.

**Figure 3:**
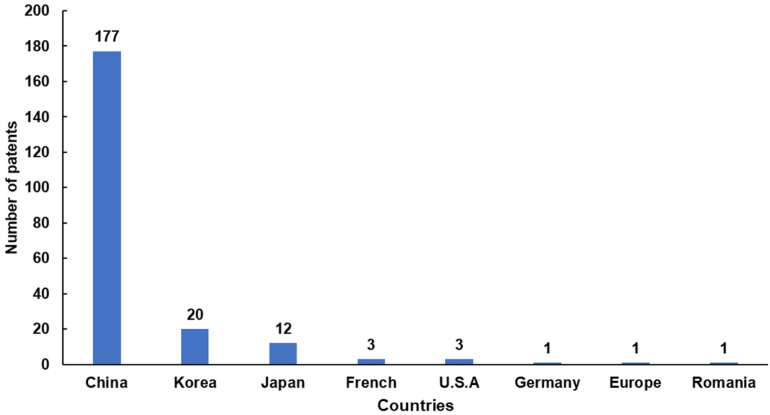
The number of applications for lily-related patents in different countries (2005–2021).

Asia, especially East Asia, is the major region for lily-related cosmetic patent applications. China has an absolute preponderance of lily-related cosmetic patent applications (177), followed by Korea (20) and Japan (12). The number of relevant patent applications in the top 3 countries accounted for 95.87% of the total (Figure 3). This implied that lilies are more likely to be used as a cosmetic raw material source in East Asia than in other parts of the world. This should stand to reason because lilies have long been used as food and as important medicine in China, Korea, and Japan [35,62,63]. The size of the patent family is one of the measures of its potential value. Generally, the larger the patent family, the wider the area it involves, and the more core technology and potential value it has [64,65,66]. The application JP2016169238A has the largest family of lily-related cosmetics patents, reaching 29, followed by TW201304819A (13), JP2012509255A (10), US20180344626A1 (9), CN103211728A (6), CN103893722A (4), CN108686107A (4), and EP2465518A1 (3), and the others contain fewer than three items (Supplementary Table S1).

### 4.3. Application and Efficacy of Lily in Cosmetics

The application of lilies covers almost all aspects of cosmetics, including facial masks, sunscreens, facial cleansers, skin creams, lipsticks, toners, face creams, makeup removers, toothpastes, shower gels, shampoos, deodorants, perfumes, and essences (Supplementary Table S1). Lily-related cosmetics also have many functions, including skin care, whitening, antioxidation, antiaging, wrinkle removal, spot lightening, acne removal, moisturizing, antiradiation, skin repair, heat clearing, hair-growth promotion, and hair darkening (Supplementary Table S1).

**Figure 4:**
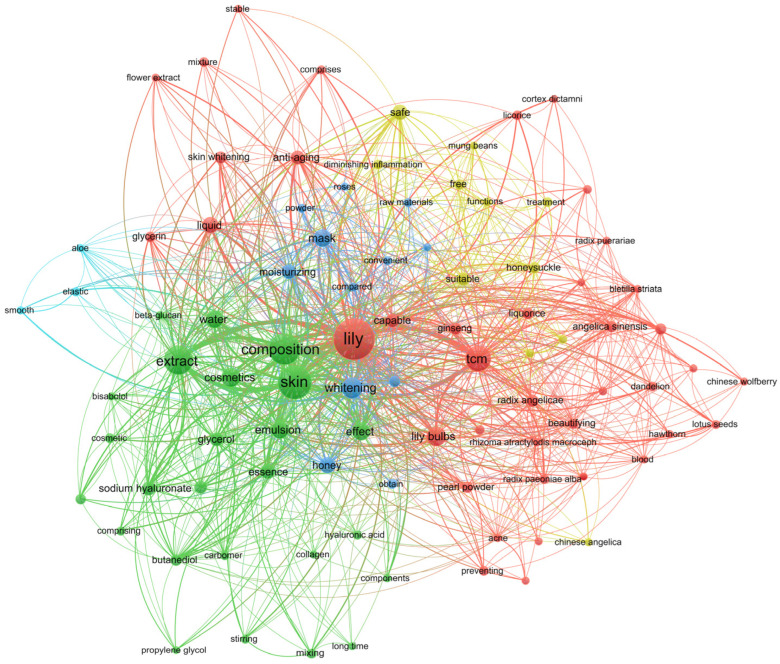
Keyword co-occurrence analysis of lily-related patents. Note: tcm indicates traditional Chinese medicine composition.

However, with the exception of a few cosmetics with lilies alone as a botanical source, the majority of patented products are mixtures consisting of ingredients from lilies and other natural sources. To further understand the efficacy and compatibility of lilies in cosmetics, we used Vosviewer to analyze the co-occurrence of relevant keywords in 218 patent abstracts. After manually removing irrelevant words and merging synonyms, we selected keywords with a frequency ≥ 5 to construct a co-occurrence relationship map (Figure 4). The results showed that the vocabulary with a higher frequency of occurrence can be divided into three main categories: raw materials, product types, and functions. Facial masks, emulsions, liquids, and essences are the most widely used cosmetics related to lilies. Cosmetics with lilies as the core connecting point have the highest correlation with the skin, including moisturizing, whitening, antiaging, beautifying, freckle-removing, acne-removing, skin-smoothing, nourishing, anti-inflammation, and blood-circulation-promoting effects. The cosmetic ingredients most compatible with lilies include honey, ginseng, honeysuckle, liquorice, angelica sinensis, pearl powder, poria cocos, dandelion, radix paeoniae alba, lotus seeds, radix astragali, aloe, bletilla striata, Chinese angelica, Chinese wolfberry, hawthorn, mung beans, peach kernel, radix puerariae, atractylodis macrocephalae, roses, and white poria. For lilies, keyword co-occurrence analysis shows that the bulbs of lilies are the most commonly used part in cosmetics, followed by flowers. Most of these components are extracts, and a few are applied in the form of powder. This suggests that most of the natural ingredients commonly used in compatibility with lilies are traditional Chinese medicine (tcm), and the keyword “tcm” is also in the high-frequency vocabulary. Moreover, “no side effects” also appeared in the high-frequency-related vocabulary, stating that the safety of a large proportion of plant-based natural products used in cosmetics is trustworthy [1,3,4,6,7].

## 5. Granted Lily-Based Cosmetics

The grant and transfer of patents reflect their potential value to some extent [64,65,66,67,68]. To gain further insight into the application areas and functions of lily-related cosmetic patents, we collated the relevant patent information that was granted (and/or transferred). A total of 102 patents have been granted, of which 36 have been transferred (one is under examination) (Table 2). This clearly shows that lily-related patents are involved in all aspects of cosmetics, including masks, emulsions, creams, toothpastes, deodorants, hair restorers, gums, toners, facial cleansers, perfumes, skincare products, eye shadow, makeup removers, shampoos, cosmetic drinks, and cosmetic traditional Chinese medicine compositions. Their functions were also quite diverse, ranging from beautification, slimming, hair-growth promotion, and hair darkening to the treatment of skin diseases (Table 2). These data suggest that the application of lilies in cosmetics is popular and has great potential.

## 6. Opportunities and Challenges

With the improvement of people’s living standards, aesthetic requirements are increasing, leading to the increasingly extensive use of cosmetics. Moreover, consumption goods derived from natural resources are increasingly being promoted because of the awareness of sustainable production and long-term health benefits [4]. Liliaceae has been reported to be one of the major plant family resources of natural-product-based cosmetics [8]. The genus *Lilium*, as an important group of Liliaceae, includes more than 100 wild species and thousands of cultivars [33]. Phytochemical studies have proven that the *Lilium* plant is rich in bioactive components such as phenolic acids, flavonoids, and polysaccharides, which are important component bases for their application in cosmetics [9,10,11,12,13]. The abundant *Lilium* species provide a wide range of material sources for their applications in cosmetics. In addition, nearly 30 species of *Lilium* plants have been used as food and medicine since ancient times [35], implying the safety of lily application in cosmetics (or that at least these species, which have been consumed and medically used, are safe for application in cosmetics). Moreover, the increasingly popular consumption concept of using oral food supplements to achieve cosmetic results from the inside out also provides an opportunity for the application of edible and medicinal lilies in cosmetic foods and phytomedicine composition [3].

However, the current applications of lilies in cosmetics are usually added as extracts, and a few as lily powder, and all are obtained from wild lily species, which limits their material sources. Therefore, it is urgent to develop new *Lilium* materials for application in cosmetics. Our previous studies have shown that some of the underutilized wild lilies and many cultivars with higher active ingredient contents may have greater potential for cosmetic applications [12]. Moreover, studies have shown that, in addition to bulbs, the other tissues of lilies are also rich in phenolics and polysaccharides, which are usually discarded as waste materials during their production. So, it is also promising to make full use of these wastes from agricultural production (e.g., leaves, roots, etc.) to extract supplements in cosmetics. However, their safety, especially as cosmetic foods and drugs, has not been evaluated. Therefore, while vigorously developing new lily resources as cosmetic ingredients, it is also a challenge to fully evaluate their safety.

## 7. Conclusions and Outlook

**Figure 5:**
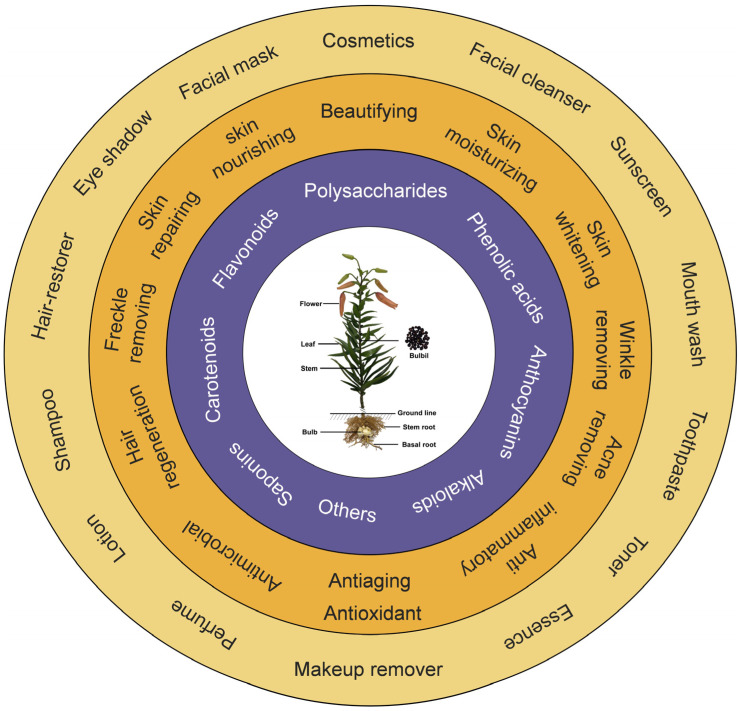
Metabolites, functions, and applications in cosmetics of *Lilium*.

*Lilium* plants are rich in health-benefit components. Among them, phenolic acids and flavonoids have strong antioxidant, anti UV radiation, and antibacterial abilities, and polysaccharides have good skin moisturizing, antibacterial, and skin repairing abilities. Other ingredients such as saponins, alkaloids, carotenoids, and anthocyanins also have potential health benefits. These metabolic components provide the material and functional basis for the application of lilies in cosmetics. Figure 5 summarizes the chemical constituents of the *Lilium* plant, and its cosmetic functions and applications in cosmetics. Currently, lilies are widely used in cosmetics, and their functions are also diverse and extensive. Additionally, most of these applications are compatible with other plant-based ingredients. Moreover, the concept “beauty from within” is a new trend that advocates beauty and slimming by oral supplementation. *Lilium*, as a traditional ethnomedicinal herb, is widely used in several countries for health products and disease treatment, highlighting its potential application in cosmetic food. However, since only a small number of *Lilium* species are currently allowed to be applied in cosmetics, this leads to a lack of its raw materials. Considering that the current applications of lilies in cosmetics are dominated by crude extracts, specific substance-related information on its role in cosmetic action is lacking. Therefore, further dissections of the functions of specific components in *Lilium* plants are necessary. Meanwhile, the development of new lilies species and cultivars, and the full utilization of waste from the agricultural production of lilies for the extraction of cosmetic raw materials, are the solutions to the shortage of raw materials for cosmetic applications, but their safety still needs to be evaluated.

## Figures and Tables

**Table 1:** Contents of total phenolic acids, total flavonoids, and antioxidant capacity in different lilies.

Specie/ Varieties	TPC	TFC	DPPH	ABTS	FRAP	CUPRAC	Reference
*L. amabile*	176.57 ± 10.65	77.14 ± 5.76	529.04 ± 11.76	314.29 ± 44.01	717.80 ± 87.93	/	[9]
*L. brownii*	245.27~283.28	95.85~122.55	578.02~660.32	507.39~522.04	801.71~896.26	/	[9]
*L. brownii* var. *viridulum*	353.42~487.29	127.30~222.69	556.47~634.85	579.3~876.28	921.56~1391.66	/	[9]
*L. brownii* var. *viridulum*	69.68	88.40	175.61	/	147.26	531.38	[12]
*L. callosum*	433.20 ± 32.87	139.02 ± 3.64	732.82 ± 35.92	757.76 ± 129.73	1291.78 ± 95.08	/	[9]
*L. cernuum*	379.64~513.45	68.40~193.49	642.68~811.19	290.32~1006.79	817.69~1636.70	/	[9]
*L. concolor*	3897.60 ± 42.54	413.45 ± 2.03	455.31 ± 7.21	1143.67 ± 11.28	/	1025.14 ± 45.68	[48]
*L. concolor* var. *pulchellum*	140.77~281.15	70.76~134.81	505.53~591.74	312.96~649.89	535.36~1217.21	/	[9]
*L. dauricum*	277.79~328.99	110.80~131.75	579.98~726.94	591.29~684.51	1089.36~1237.18	/	[9]
*L. davidii*	181.04 ± 14.46	68.70 ± 4.08	525.12 ± 60.04	352.91 ± 54.24	910.91 ± 55.93	/	[9]
*L. davidii*	87.45	248.92	418.65	/	291.67	842.13	[12]
*L. davidii* var. *unicolor*	2017.17 ± 140.20	150.53 ± 3.66	404.48 ± 14.59	848.49 ± 9.17	/	595.61 ± 7.24	[48]
*L. davidii* var. *willmottiae*	249.12~331.10	91.35~137.37	558.43~726.94	558.00~647.22	1097.35~1185.24	/	[9]
*L. davidii* var. *willmottiae*	188.17	258.28	822.23	/	427.91	1525.78	[12]
*L. distichum*	292.20~393.06	86.23~144.13	568.23~779.84	338.26~655.21	659.21~1327.74	/	[9]
*L. formosanum*	231.47 ± 10.55	79.10 ± 3.19	621.13 ± 33.43	354.24 ± 39.41	857.64 ± 36.25	/	[9]
*L. henryi*	1040.13~1605.25	382.99~898.61	768.09~1089.43	1591.42~2353.17	3310.69~7024.90	/	[9]
*L. henryi*	1162.79	1222.24	5293.75	/	3490.72	10,624.99	[12]
*L. lancifolium*	328.11~568.80	105.98~492.16	597.62~787.68	683.18~1492.88	1410.31~2479.69		[9]
*L. lancifolium*	225.45	435.58	896.03	/	490.67	1289.39	[12]
*L. lancifolium*	2827.25 ± 55.50	227.24 ± 3.66	541.27 ± 3.43	1075.51 ± 2.94	/	842.04 ± 8.32	[48]
*L. leichtlinii* var. *maximowiczii*	96.06~200.33	75.57~80.73	570.19~572.15	242.37~327.61	298.31~716.47	/	[9]
*L. leichtlinii* var. *maximowiczii*	94.08	250.93	375.21	/	277.38	806.62	[12]
*L. leucanthum*	1101.95~1666.05	298.15~710.28	724.98~909.17	1389.00~2229.32	2831.27~5259.03	/	[9]
*L. leucanthum*	2336.00 ± 29.28	521.19 ± 17.77	507.64 ± 6.85	889.38 ± 13.42	/	799.34 ± 5.81	[48]
*L. leucanthum* var. *centifolium*	1158.74~1496.80	562.50~959.47	779.84~1011.05	1840.46~2213.34	3461.18~5175.12	/	[9]
*L. pumilum*	267.83~518.09	104.13~174.45	538.84~630.93	410.17~888.27	788.39~1427.62	/	[9]
*L. pumilum*	4177.39 ± 57.19	339.13 ± 9.17	546.51 ± 9.77	1091.96 ± 5.70	/	1044.10 ± 11.30	[48]
*L. regale*	1548.68~2014.82	633.18~1304.39	979.70~1293.20	2289.25~2531.63	5534.69~10,850.98	/	[9]
*L. regale*	1373.43	1591.28	6675.08	/	4643.63	12,906.79	[12]
*L. regale*	10,381.49 ± 49.12	1428.21 ± 38.52	600.33 ± 2.24	1173.28 ± 11.41	/	1438.01 ± 16.56	[48]
*L. rosthornii*	813.14~1123.96	304.58~452.35	805.31~848.42	1475.56~1949.66	3051.01~4121.72	/	[9]
*L. sargentiae*	1121.87~1797.40	301.06~763.80	793.56~1091.39	1639.37~2362.50	3066.98~7159.41	/	[9]
*L. sulphureum*	1442.60~1807.20	668.34~860.56	830.79~1073.75	1936.34~2387.80	4846.19~8433.88	/	[9]
*L. taliense*	1056.74 ± 14.27	542.35 ± 10.65	779.84 ± 13.57	1791.18 ± 115.54	2923.16 ± 62.70	/	[9]
*L. tsingtauense*	392.92 ± 3.24	110.12 ± 8.83	630.93 ± 38.25	543.35 ± 76.22	957.52 ± 60.11	/	[9]
*L. wenshanense*	280.05 ± 8.43	63.67 ± 6.56	791.60 ± 13.58	194.43 ± 48.16	475.43 ± 41.52	/	[9]
*L martagon*	/	/	245.82 ± 1.59 (IC50)	147.42 ± 1.93 (IC50)	/	/	[13]
*L pumilum*	/	1.04% DW	/	/	/	/	[10]
*L.* ‘Amiga’	57.05	164.82	309.91	/	188.08	549.14	[12]
*L.* ‘Ceb Dizzle’	110.53	184.81	422.24	/	233.50	584.65	[12]
*L.* ‘Dandie’	72.23	216.50	426.67	/	270.23	571.34	[12]
*L.* ‘Ercolano’	58.65	131.16	276.76	/	166.14	433.72	[12]
*L.* ‘Franson’	359.63	247.57	1624.76	/	926.38	2936.37	[12]
*L.* ‘Jinghe’	59.89	179.81	278.15	/	171.25	522.51	[12]
*L.* ‘Red Life’	80.81	165.26	378.58	/	231.46	602.41	[12]
*L.* ‘Red Velvet’	95.07	171.25	443.83	/	276.36	677.88	[12]
*L.* ‘Robina’	316.27	290.63	1094.31	/	573.84	2666.68	[12]
*L.* ‘Siberia’	394.56	473.41	1886.23	/	1251.49	3221.59	[12]
*L.* ‘Sobonna’	321.34	378.86	1382.33	/	841.51	3243.79	[12]
*L.* ‘Tarrango’	420.65	519.10	1986.49	/	1246.26	4233.75	[12]
*L.* ‘Terrasol’	365.29	384.96	1693.52	/	969.46	3194.96	[12]
*L.* ‘White Heaven’	181.75	312.59	892.25	/	421.27	1432.56	[12]
*L.* ‘Zuma’	283.35	283.39	1228.04	/	682.22	2413.64	[12]
*L.* Varieties	144.51~340.55	97.73~456.82	0.530~2.373 (IC50)	/	/	/	[13]

Note: TPC: total phenolic acid content; TFC: total flavonoid content; DPPH: 1, 1-Diphenyl-2-picrylhydrazyl radical 2, 2-Diphenyl-1-(2,4,6-trinitrophenyl) hydrazyl radical scavenging ability; ABTS: 2, 2′-azino-bis (3-ethylbenzothiazoline-6-sulfonic acid) radical scavenging ability; FRAP: ferric ion reducing antioxidant power; and CUPRAC: cupricion reducing antioxidant capacity. Unless specified noted, TPC is expressed in mg (gallic acid equivalent)/100 g dry weight; in [12], TFC is expressed in mg (quercetin equivalent)/100 g dry weight; TFC in other references is expressed in mg (rutin equivalent)/100 g dry weight; in [9], DPPH is expressed in µmol (gallic acid equivalent)/100 g dry weight, and in other references, it is expressed in µmol (trolox equivalent)/100 g dry weight; and ABTS, FRAP, and CUPRAC are expressed in µmol (trolox equivalent)/100 g dry weight. “/” indicates not determined.

**Table 2:** Applications and main functions of the granted lily-related cosmetic patents.

Publication Number	Application Area	Functions
CN1814216A	TCMC	acne removal
CN101032599A	liquid	skin nourishment, beautification, skin care, and slimming
CN101125119A	cosmetic	skin whitening, antiaging
KR1020100079826A	cosmetic	anti-radiation
CN101658518A	hair restorer	promotion of hair regeneration, normalization of follicle structure
JP2012509255A	cosmetic	antioxidant
CN101904982A	TCMC	treatment of rosacea, as an anti-inflammatory, acne removal, and freckle removal
CN101884610A	mask	skin whitening, freckle removal, skin moisturizing, and skin rejuvenation
CN101879281A	gum	freckle removal, as a treatment for alopecia, and to turn white hair into black hair
CN102145123A	cosmetic	speckle removal
JP2011225564A	cosmetic	skin whitening, beautification
CN102166288A	TCMC	to treat juvenile canities, melanogenesis promotion
CN102166313A	/	for curing tinea pedis, skin care
CN102309750A	film	as an antibacterial, as an anti-inflammatory, and for skin whitening
CN102178637A	mask	/
CN102228612A	TCMC	for treating tinea manus
CN102247523A	electuary	beautification
CN103211728A	cream	beautification
CN103211743A	toner	skin moisturizing, skin whitening, skin rejuvenation
CN103222945A	liquid	skin care, skin moisturizing, and skin whitening
EP2465518A1	cosmetic	skin whitening
TW201304819A	cosmetic	skin whitening
CN102512616A	TCMC	acne removal
CN102552748A	TCMC	beautification, skin rejuvenation
CN102525874A	facial cleanser	freckle removal, skin moisturizing, skin whitening, skin rejuvenation, wrinkle removal, clearing blackheads
CN102579908A	TCMC	skin care, skin rejuvenation, minimizing pores, and skin repair
CN102614368A	beverage	skin nourishment, freckle removal
CN102861269A	TCMC	acne removal
KR1020140004463A	cosmetic	skin care
CN102920646A	emulsion	skin moisturizing and nourishing
CN102961586A	TCMC	treating tinea
CN102961319A	sunscreen	skin care, anti-radiation
CN103054746A	essence	skin care, skin whitening, freckle removal
CN103156801A	toner	skin moisturizing, wrinkle removal, skin smoothing, freckle removal, skin softening, and beautification
CN103372136A	TCMC	treating favus of the scalp
CN103385844A	/	antioxidant, anti-allergy, and antiaging
CN103463570A	TCMC	treating alopecia
CN103520666A	TCMC	promoting blood circulation, beautification
CN103550511A	TCMC	anti-aging
KR1020150056184A	perfume	/
CN103637974A	bath bag	promotion of hair regeneration, skin care, and slimming
CN103655446A	/	beautification
CN103655447A	/	antiaging, freckle removal
CN103690798A	TCMC	promotion of hair regeneration
CN103736083A	/	beautification, slimming
CN103893722A	TCMC	anti-radiation
CN103919707A	cosmetic	skin whitening, freckle removal
CN103989601A	cream	skin whitening, skin moisturizing, and skin care
CN104013563A	essence	freckle removal, skin whitening
CN104013851A	TCMC	treating beriberi
CN104083317A	cream	skin care, antiaging, pouches removal, and black-eye removal
JP2016069332A	cosmetic	elastin production promotion, antiradiation
FR3026946A1	cosmetic	skin whitening, skin care
CN104306308A	makeup remover	skin moisturizing, skin cleaning
CN104352944A	TCMC	acne removal
CN105770377A	cosmetic	skin care, skin moisturizing
JP2016121113A	cosmetic	anti-photoaging, turning white hair into black hair
CN104490712A	TCMC	skin nourishment, antiaging
KR1020160114794A	cosmetic	skin care, skin moisturizing, and wrinkle removal
JP2016199487A	deodorant	deodorization
CN104771340A	mask	antiaging, freckle removal
CN104922004A	cosmetic	skin moisturizing, skin care
CN105147543A	hydrogel	skin whitening
CN105168059A	toothpaste	cleaning oral cavity
CN105148244A	TCMC	treating urticaria
US20180344626A1	cosmetic	skin cleaning
CN105326754A	mask	acne removal, antibacterial, and anti-inflammatory
CN105326755A	mask	acne removal
CN105381335A	lotion	treating eczema
CN105687093A	cosmetic	antiaging, skin care
CN105943466A	shampoo	hair care, skin moisturizing
JP2016169238A	cosmetic	skin whitening, wrinkle removal
CN106309351A	cosmetic	skin whitening
CN106421487A	TCMC	skin repairing
KR1020180064146A	perfume	/
CN109223973A	TCMC	anti-radiation
CN107320395A	eye shadow	skin moisturizing, skin repairing, skin whitening, and wrinkle removal
KR1020190051450A	cosmetic	skin care, skin moisturizing
CN108078879A	cosmetic	acne removal, anti-inflammatory, anti-radiation, antioxidant
CN108066237A	cosmetic	skin whitening, freckle removal, skin care
CN108434319A	TCMC	wrinkle removal, and skin moisturizing
CN108686107A	TCMC	promotion of hair regeneration
CN108567891A	TCMC	dredging pores, skin cleaning, and acne removal
CN108434069A	cosmetic	skin moisturizing, skin repairing
CN108653137A	cosmetic	skin care, skin whitening, antioxidant, and skin repairing
CN108815077A	cream	skin care, skin moisturizing
CN108852869A	cosmetic	anti-radiation, skin care
CN109044935A	makeup remover	skin repairing skin moisturizing
CN109199988A	cosmetic	skin whitening, wrinkle removal, skin moisturizing, and skin rejuvenation
CN109394959A	TCMC	skin repairing, skin care
CN109432290A	TCMC	anti-allergy
KR1020200079139A	/	antioxidant
CN109646359A	mudpack paste	skin cleaning, beautification
KR1020200134929A	/	wrinkle removal, skin whitening, and anti-inflammatory
CN110251425A	perfume	/
CN110368355A	eye cream	antiaging, antioxidant, and wrinkle removal
CN110507752A	TCMC	acne removal
KR1020200068575A	cosmetic	wrinkle removal, skin whitening, and anti-inflammatory
CN112043637A	essence	skin repairing
CN112107505A	shampoo	turning white hair into black hair
CN112057359A	cosmetic	skin moisturizing
KR1020200138142A	cosmetic	skin care, skin moisturizing, and wrinkle removal
US20210154132A1	cosmetic	skin care

Note: TCMC in the application area indicates traditional Chinese medicine composition; cosmetics indicate no specific scope of application specified. “/” indicates not specified.

## Supplementary Materials

The following supporting information can be downloaded at: https://www.mdpi.com/article/10.3390/antiox11081458/s1, Supplementary Table S1: Detailed information of lily-related cosmetic patents.
